# A197 ACUTE ILEITIS IN ADULT PATIENT: A RETROSPECTIVE STUDY OF PATIENT OUTCOMES

**DOI:** 10.1093/jcag/gwab049.196

**Published:** 2022-02-21

**Authors:** M Bachand, A Paradis

**Affiliations:** gastroenterology, CHUS, Sherbrooke, QC, Canada

## Abstract

**Background:**

Rising availability of medical imaging exams in the emergency room potentially leads to an increase of ileitis findings by imaging in a context of gastrointestinal symptoms. However, it can be difficult to determine the clinical significance of this radiological finding in an acute consultation setting and to establish the relevant investigations.

**Aims:**

The aim of this study is to describe the current management and the long-term outcome of patients with diagnostic of acute ileitis on imaging during an emergency consultation in our center, Centre Hospitalier Universitaire de Sherbrooke(CHUS).

**Methods:**

We performed a retrospective study of all patients diagnosed with ileitis by abdominal ultrasound or computed tomography (CT scan) during a visit in our emergency department over the years 2010 and 2017. Patients with previous diagnosis of Crohn disease were excluded. Data collected were clinical, radiological and endoscopic at diagnostic and over at least 3 years following the initial visit.

**Results:**

A total of 118 files were reviewed. The initial imaging modality was 43% ultrasound and 57% CT scan. 36% of patients had an ileocolonoscopy within one month of the initial visit. Of these, 60% demonstrated macroscopic endoscopic abnormalities. 51% of patients had follow-up imaging within 3 months of the initial consultation. 64% of these images demonstrated persistence of pathological changes in the ileal region. Only 48% had a stool culture at diagnosis. 72% of patients met a gastroenterologist and 45% saw a general surgeon. The most common cause of ileitis was an infectious origin at 44% predominantly with *Yersinia enterocolitica* and *Campylobacter jejuni*. 13% received a diagnostic of Crohn disease. 20% of ileitis remained undetermined. Among these, there was a case of Crohn’s disease which was eventually diagnosed 2 years later. The initial factors associated with a diagnosis of Crohn’s disease were family history of inflammatory bowel disease (OR 6.17 95% CI 1.67 to 22.781 p = 0.006), extra-digestive manifestations (OR 23.31 95% CI 2.255 to 240.92 p = 0.008), discontinuous impairment at initial imaging (OR 19.8 95% CI 4.284 to 91.52 p = 0.000), presence of stenosis at initial imaging (OR 14.429 95% CI 1.227 to 169.68 p = 0.034), the presence of abscess or collection on imaging (OR 11.54 95% CI 1.76 to 75.63 p = 0.011) and wall thickness in mm (1.22 95% CI 1.06 to 1.40 p = 0.005).

**Conclusions:**

Our study shows that the majority of acute ileitis found on imaging are of infectious origin. A portion of acute ileitis corresponded to the initial presentation of Crohn’s disease. Thus, we believe that it is preferable to offer at least clinical follow-up to patients with acute ileitis on imaging and to consider additional investigation by imaging and / or colonoscopy when symptoms persist and this particularly in presence of factors associated with Crohn’s disease.

**Funding Agencies:**

None

